# The encounter with God in myth and madness

**DOI:** 10.1186/1747-5341-2-12

**Published:** 2007-07-03

**Authors:** Otto Doerr, Óscar Velásquez

**Affiliations:** 1 Department of Psychiatry at the Institute of Psychiatry, University of Chile: Av. La Paz 841, Santiago, Chile; 2 Department of Ancient Philosophy, University of Chile: Av. Capitán Ignacio Carrera Pinto 1025, Santiago, Chile; 3 Faculty of Medicine, University “Diego Portales”, Gorbea 1770, Santiago, Chile

## Abstract

**Background:**

It is well known how often psychiatric patients report religious experiences. These are especially frequent in schizophrenic and epileptic patients as the subject of their delusions. The question we pose is: are there differences between this kind of religious experiences and those we find in religious texts or in the mythological tradition?

**Results:**

An overview on famous mythological narratives, such as The Aeneid, allows us to establish that the divinities become recognizable to the human being at the moment of their departure. Thus, Aeneas does not recognise his mother, Venus, when she appears to him in the middle of the forest at the coast of Africa. A dialogue between the two takes place, and only at the end of the encounter, when she is going away and already with her back to Aeneas, she shows her son the signs of her divinity: the rose-flush emanating from her neck, her hair perfume and the majesty of her gait. Something analogous can be observed in the encounter of Moses with Yahweh on Mount Sinai. Moses asks God: "Show me your glory, I beg you". And God replies, among other things: "you shall see the back of me, but my face is not to be seen". In the same sense, the Emmaus disciples do not recognise Jesus till the moment of his disappearance ("but he had vanished from their sight"), and Saul of Tars falls off his horse just in the moment when he feels the divine presence. In short, the direct encounter with the divinity seems not to occur in the realm of myth or in religious tradition. The realm of madness is exactly the opposite. Our research on religious experiences in schizophrenic and epileptic patients leads us to conclude that God appears to them face to face, and the patient describes God the father, Jesus or the Virgin Mary in intimate detail, always in an everyday setting. So, the divinity is seen in the garden, or in the bedroom, or maybe above the wardrobe, without any of its majesty. The nearness to God also tends to be so extreme that even an identification of patient and God can occur. That light emanating from the world of the divine ceases to be perceived by them.

**Conclusion:**

While in mythological narratives God appears to the human being at the moment of His departure or showing His back, psychiatric patients with religious delusions experience the divinity in a direct way, face to face. Given the deformation of the divine occurring on the edge of madness we can better understand the mysterious words from Yahweh to Moses in Exodus: "for man cannot see me and live".

## Exposition

The fact that both in literary Greco-roman tradition and in the religious texts of the Semitic tradition the divinity or divinities make themselves recognizable to the human being at the moment of their departure and not at their arrival is remarkable. A very graphic example of the above is the appearance of Venus to her son Aeneas in the middle of a forest at the coast of Africa, where the hero had arrived after surviving a tempest. Aeneas explores the surroundings and before he even realizes where he is, his mother, the goddess Venus, appears before him, "disguised as a maiden in face and dress, with a girl's weapons – a Spartan girl... In huntress wise she had handily slung her bow from her shoulders, and her hair was free to blow in the wind, and she went bare-kneed with the flowing folds of her dress kilted up and securely knotted." [1, p. 88]. Aeneas does not recognize her, and at her salute, he answers saying: "No sight or sound have I had of any of your sisters, o-but what shall I call you, maiden? for your face is immortal, and your speech rings not of humankind...". And she answers with apparent humility: "Believe me, such titles are not my due: it is the fashion for Tyrian girls to carry a quiver and wear like this the high-laced, crimson hunting boot..." [1, p. 89]; he then goes on to describe to him the country where he has ended up, and to point out to him the road to Queen Dido, assuring him that he will recover his companions and his "disbanded fleet". At the end of the encounter, when she is ready to go away, and **already with her back to Aeneas, **she shows her son the signs of her divinity, the same that are going to allow Aeneas to recognize her: 1) the "rose-flush" emanating from her neck *(rosea cervice refulsit)*, 2) her hair perfume (the poet says: "her crown of ambrosial hair breathed out a heavenly fragrance", [1, p. 90], and 3) the majesty of her gait *(et vera incessu dea)*. That is to say, the supernatural grace of the goddess is revealed just as Anaeas studies his mother's traits. The text continues narrating how he "sent these words in her wake: – Must you too be cruel, Must you make game of your son with shapes of sheer illusion? Oh, why may we not join hand to hand, or ever converse straightforwardly?" [1, p. 90–91]. In summary, Aeneas is not allowed to encounter his goddess-mother face to face, nor join his hands with hers, as any son would like. He is permitted to recognize her only in the moment of her departure, when she turns her back on him, which is the precise moment that she reveals her divine splendour.

We observe something analogous in the encounter of Moses with Yahweh on Mount Sinai. God has already given Moses the order to depart to the Promised Land. Moses must climb the mountain with the new engraved tables, and before doing so he says to Yahweh: "...please let me know your ways, so I can understand you and win your favour. Remember, too, that this nation is your own people." [2, Exodus 33, 13]. And God answers him: "I myself will go with you, and give you rest." Moses says, "If you are not going with us yourself, do not make us leave this place. By what means can it be known that I, I and my people, have won your favour, if not by your going with us? By this we shall be marked out, I and my people, from all the peoples on the face of the earth.' Yahweh said to Moses, 'Again I will do what you have asked, because you have won my favour and because I know you by name'." [2, Exodus 33, 13–17] When hearing these kind words from the Lord, Moses asks him what any son would ask his father or any mistress her beloved: to see him. Thus, he says, "Show me your glory, I beg you". And God replies: "I will let all my splendour pass in front of you, and I will pronounce before you the name Yahweh. I have compassion on whom I will, and I show pity to whom I please. You cannot see my face... for man cannot see me and live.' And Yahweh said, 'Here is a place beside me. You must stand on the rock, and when my glory passes, I will put you in a cleft of the rock and shield you with my hand while I pass by. Then I will take my hand away and **you shall see the back of me **(underlined by this author); but my face is not to be seen."' (Exodus 33, 18–23). Again we are looking at the same phenomenon observed in the relationship of Aeneas with Venus: when God, the superior and transcendental being, manifests himself, He/it can be beheld only from behind, when His back is turned, already in the process of leaving. This biblical scene was often commented on by the Fathers of the Church, in particular by Saint Agustin [3]. According to him, the image of God -essentially an invisible being – is possible only at God's whim, who manifests himself to whomever He wishes, however He pleases, and under the appearance He wants, even while "His nature remains invisible". Moses, by contrast, being a good pious man of his time, as a good son, wanted to see his father God *in natura propria *(as He really was). Saint Agustin concludes his comments saying: "No living being can see Him in this life such as He is. Many have seen what the divine will has decided to show, but not what human nature desired." And Saint Gregory of Nice, for his part, interprets this passage as Moses' constant ascent toward the heights, attracted by God's back, and aspiring to "satisfy himself in the archetype itself" [4].

The case of Jesus' encounter with the disciples in Emmaus also represents an analogy to above narratives. The text is as follows: "That very day, two of them were on their way to a village called Emmaus, seven miles from Jerusalem, and they were talking together about all that had happened (Passion and Death of Jesus Christ). Now as they talked this over, Jesus himself came up and walked by their side, **but something prevented them from recognizing him **(underlined by this author). He said to them, 'What matters are you discussing as you walk along?' They stopped short, their faces downcast. Then one of them, called Cleopas, answered him, 'You must be the only person staying in Jerusalem who does not know the things that have been happening there these last few days'." [2, Lk 24, 13–18]. They walk with Him the whole way without recognizing Him, while the Master explains to them the meaning of the Scriptures and how all these events had been already foretold by the prophets, "beginning with Moses". At nightfall the Emmaus disciples ask the outsider to stay with them. They did not recognize Him either at the beginning of the supper, but when "...He took the bread and said the blessing; and He broke it and handed it to them." [2, Lk 24, 30]; only then "...their eyes were opened and they recognized him" [2, Lk 24, 31]. Nevertheless, the text proceeds, immediately afterwards, "...but he had vanished from their sight." [2, Lk 24, 31]. The eye-opening and recognition of the object is perfectly synchronized with its final fading away, to such extent that it is not clear if it is actually the object that disappears, or if it is the subject which, when becoming aware of the divine presence, dissipates, so to speak, the figurative representation, and retains only that superior reality, which remains beyond his grasp. The total simultaneity of the two sensations appears psychologically incompatible. There is a fleeting instant when the creature understands; and that is the same instant when the figure disappears. The Emmaus disciples should have recognized Jesus during the long road they travelled together, through his clarifying words, which could have come only from the One who had been the very victim of the phenomena that so concerned them; but there the Lord was too close: it was an encounter face to face, and they can recognize them only in the moment when He withdraws, when He vanishes from their sight. They themselves became aware of this strange failure to recognize him when, at the moment the presence of Jesus vanishes, "...they said to each other, 'Did not our hearts burn within us as He talked to us on the road and explained the scriptures to us?"' [2, Lk 24, 32].

Once Jesus has disappeared, there comes for the Emmaus disciples the sacramental effect by which they get the courage to go to Jerusalem, and there they bear witness to having seen Jesus. And the text proceeds: "...they found the Eleven assembled together with their companions, who said to them, 'Yes, it is true. The Lord has risen and has appeared to Simon.' Then they told their story of what had happened to them on the road and how they had recognised him at the breaking of bread." [2, Lk 24, 33–35]. Thus, God appears in a privileged moment saved by the scriptures from being forgotten. Once it is transformed in narrative it takes on the characteristics of a revelation, and thus, the theophany acquires a literary dimension. The initiative is now in the hands of the creature, who by means of a movement to approach the divinity re-establishes contact between man and that God proclaimed by the discourse and favourably disposed to those who seek Him; and Christianity offers the peculiarity that the discourse, **the logos itself, becomes flesh, **that is to say, it is unified and incarnated in the person of Christ. The normal road will then be meditation and prayer, and the hope of ever achieving a direct encounter, face to face with divinity, will have to be forsaken.

An extreme example of the impossibility of a face to face encounter with God is what happens to Paul of Tars, persecutor of Christians, on the road to Damascus. Let us remember narrative from the Acts of the Apostles: "Meanwhile Saul was still breathing threats to slaughter the Lord's disciples. He had gone to the high priest and asked for letters addressed to the synagogues in Damascus that would authorise him to arrest and take to Jerusalem any followers of the Way, men or women that he could find. Suddenly, as he was travelling to Damascus and just before he reached the city, there came a light from heaven all around him. He fell to the ground, and then he heard a voice saying, 'Saul, Saul, why are you persecuting me?' 'Who are you, Lord?' he asked, and the voice answered, 'I am Jesus, and you are persecuting me. Get up now and go into the city, and you will be told what you have to do. The men travelling with Saul stood there speechless, for though they heard the voice they could see no one. Saul got up from the ground, but even with his eyes wide open he could see nothing at all, and they had to lead him into Damascus by the hand. For three days he was without his sight, and he took neither food nor drink." (Acts 9, 1–9). The divine presence, its light, blinds Saul and throws him from his horse to the ground. It is not, obviously, a light directed at the senses, but, as Tellenbach says, an atmospheric emanation from the absolutely other, from the divine You, You whose presence, whose nearness, we would add, is unbearable. The great German-speaking poet, Rainer Maria Rilke [5], describes this same irresistible proximity of divinity when in the first stanza of the First Duino Elegy he says, referring to the angels:

"And if I cried, who'd listen to me in those angelic

orders? Even if one of them suddenly held me

to his heart, I'd vanish in his overwhelming

presence. Because beauty's nothing

but the start of terror we can hardly bear,

and we adore it because of the serene scorn

it could kill us with. Every angel's terrifying."

And with this same phrase with which he finishes the first stanza of the first elegy begins the second: "Every angel's terrifying". This means that the divine, the transcending, is unbearable in its, at least visual, proximity. Analogous has been said by the most renowned of the German poets, Friedrich Hölderlin [6], in his famous poem, Bread and Wine: "humans can endure the fullness of the gods only at times" (translated by James Mitchell).

The reaction to the sound emanating from the divine force is different. So it is that Paul of Tars, on his way to Damascus, is able to listen to the voice of God and answer Him, without being destroyed. On the other hand, **his vision blinds him, **and this inability to see, as we can read in The Acts of the Apostles, lasted several days. Years later, transformed into an apostle and one of the major promoters of the new religion, Saint Paul writes an Epistle to the Corinthians, famous because he develops in it the theme of love as caritas and where, at one point, he declares: "Now we are seeing a dim reflection in a mirror ("in enigma, says another translation); but then we shall be seeing face to face. The knowledge I have now is imperfect; but then I shall know as fully as I am known." [2, I Corinthians 13, 12]. The apostle puts into words the thesis we have been developing since the beginning of this essay, that is, that in this world the knowledge of God (the greatest and deepest of truths) can be given in only a veiled, indirect way, with the back turned, as occurs to Aeneas with his god-mother Venus, "in enigma", as the epistle says. The direct knowledge of God will be possible only in the next life, where we will be able to see God such as He is ("then I shall know as fully as I am known"). Finally, in light of the foregoing, I would like to cite another tremendous verse by Hölderlin [7], which says: "The monstrous is to want to match God and man... and that can be purified only through an endless splitting"... (translated by this author).

The philosopher and mystic Plotinus often speaks of "visions" and this seems to contradict the findings we have pointed out. Nevertheless, it is necessary to keep in mind that Plotinus did not believe in a personal god. His divinity was rather nearer to Plato's idea of Beauty than to the god of the monotheistic religions. In Enneads IV, 8, describing the highest level of mystical experience, Plotinus [8] said that he sees a "marvelous beauty", which he experiences as a divinity. But to identify oneself with such an abstract concept is not the same as a direct, face to face encounter with a personal god. It is very likely that he does not mean an optical perception when he speaks of visions. We found this explained in the Fifth Ennead, when he says: "To see the divine as something external is to be outside of it; to become it is to be most truly in beauty: since sight deals with the external, there can here be no vision unless in the sense of identification with the object" [9]. Besides, Plotinus specialist, J. M. Rist [10], refers to this same point in his book *Plotinus: the road to reality*, when he writes about "the inadequate language of vision", that Plotinus "frequently employs", because what Plotinus wants to express when he speaks of "visions" is rather an "intuitive knowledge" reached by the soul, a being surrendered by "the always present One".

In any case, we find similar statements in the most famous mystics of the Spanish tradition: Saint John of the Cross and Saint Theresa of Ávila. In the first stanza of the "Spiritual Canticle" of Saint John of the Cross [11] we already read about the experience of this continuing disappearance of God:

"Where have you hidden,

Beloved, and left me moaning?

You fled like the stag

after wounding me;

I went out calling you, but you were gone".

On the other hand, Theresa of Ávila [12], in the *Seventh Mansion*, warns about the perils of false visions and contemplations, because they can be due to "our own imagination or the devil's fraud".

In today's secularised society, the experiences characteristic of religion and faith, the true encounters with God have become more and more scarce; but there is an environment in which the experience of the sacred is kept alive, namely madness. Both in schizophrenia and in manic episodes with psychoses and especially in epileptic psychoses, delusional ideas with mystic content are highly frequent [13,14].

For people with these illnesses, what is the encounter with God really like? Is it similar to that described by mythological narratives and biblical texts? And if not, then what is the difference?

In my own psychiatric practice, as chief of the ward for acute patients at the National Institute of Psychiatry in Santiago for decades, I have seen a lot of psychotic patients with religious and/or mystical experiences. I have not measured the frequency of these experiences in patients suffering from schizophrenia, but indeed in a series of twenty epileptic patients with psychosis (schizophrenia-like psychosis in epilepsy) I have personally examined and followed. Twelve of them, that is to say, 60%, showed clearly delusions with religious content. The fact of a concrete and direct encounter with the divine among both schizophrenic and epileptic patients, and this in a regular way, has deeply called our attention. With the aim of illustrating this question we will narrate in a very succinct way two of these cases: the first is the one of a schizophrenic female patient who is in treatment with me at present; the second is an epileptic patient who developed a very pronounced paranoid psychosis, whom I treated some years ago.

The study of individual cases in order to make deductions of a general validity about a given case or illness goes back a long way in psychiatry. Let's remember, by way of example, the casuistic analyses by Sigmund Freud [15] and in particular the Schreber case, or the biographical analyses of schizophrenic patients done by Binswanger [16], the best known of which is called "Ellen West". To a certain extent, medicine has always been "casuistic", only in the 20^th ^century managing to overcome that stage, thanks to the extraordinary development of new techniques of exploration of an almost incredible accuracy (e. g., brain magnetic nuclear resonance). These techniques, being able to measure the underlying anatomical and physiological disturbances we call disease, have largely shelved analyses of individual cases, at least as a source of scientific knowledge. In psychiatry, however, this type of analysis is not only legitimate, but indispensable. In the first place, because the great majority of psychopathological syndromes lack an organic basis. In the second place, because the paradigmatic cases represent real "experiments of nature", as Blankenburg says [17], in a way analogous to those that have to be "constructed" by empirical sciences to make it possible to discover their laws.

The first case refers to Teresa E., a 50-year-old woman, married, upper middle class, with grown-up children, and whose main activity is to give unpaid Religion classes in schools in poor districts. Before getting married she had a paranoid psychotic episode, from which she largely recovered. Afterward, she led a relatively normal life, although her marriage leaves much to be desired, as her husband is a materialistic and practical person, while she has always been very religious and a little removed from active social life. One day Teresa had difficulties with the pupils of a school because they laughed at her, and this forced her to make a decision between leaving that job that seemed too hard for her, and fulfilling a task that she considered entrusted to her by God Himself. This situation leads her to a state of great anxiety, and then to the apparition of hallucinatory phenomena that caused her to lose her senses and required her hospitalisation. Now calmed down, with the Neuroleptics treatment, she was able to tell me what happened: "I did not want to go on working for the Lord because of everything that had happened to me in the school, and because I felt some tugs in my head, and was very nervous, **but He showed himself to me in all his presence **(underlined by this author), causing an immense feeling of peace in my whole being. Then I went back to school to prepare children for the First Communion, and suddenly I began to feel that He caressed my heart. It was such an agreeable feeling that it cannot be explained in words." When I asked her if besides this presence of Jesus in her heart she had seen him directly, she answered the following: "On two occasions I saw him, beautiful as He is. One day I was in bed and I saw him standing there in the garden near the swimming pool, looking at my eyes, with long hair and his hands a little clenched, like a little rabbit. Another day, I was reading the book *Camino *(Way), by Monsignor Escrivá de Balaguer, and I saw Him there standing filling the doorway to my room, but in black and white, without colours. He looked at me very calmly, in a normal way, but I wondered who I am for Him to pay attention to me. This happened a long time ago, long before this crisis in school, but since then, since I saw him that I felt him nearer and nearer, until He began to give those caresses to my heart."

The second case is of Ricardo Jesus B., a 26-year-old peasant when he came to us, who has suffered since he was 16 years old from generalized epileptic seizures, which the physicians of the northern city where he went for treatment were unable to control. Fourteen months before entering the Psychiatric Hospital for a paranoid psychosis, Ricardo Jesus suffered a *status epilepticus *that lasted several hours. Once that series of seizures was over, the patient developed a psychotic condition, but no longer had attacks even after he stopped taking his medication. His delusion consisted of the following: he said that the epileptic Ricardo had died during the seizure, and that Jesus had survived, and that his mission on Earth was to do the good. He, Jesus B., had been in heaven; seated at the right of the Father, where he had felt very comfortable and been able to prove that there all was made of gold. It is true that he was also tempted by the devil, but he was able to hold out. He thinks that the physicians cannot do anything for him, because the sick person was not Jesus, but Ricardo, whom everybody in the village laughed at for his attacks, but who was now dead. Today Jesus survives, who was not identical to Jesus Christ, but was certainly very close to him, especially as he bears the same name... etc. In spite of the treatment with high doses of neuroleptics, the patient did not alter his delusion at all. However, he did not have seizures again; it could be said that he replaced the seizures with madness. In the hospital ward he was always in a good mood and became respected by the other patients. One day he asked for the certificate of discharge so politely and with irreproachable behaviour, that we let him leave. He came back to visit us twice: always glad, emanating a naive saintliness and telling anyone who wanted to hear him how happy he was to no longer be an epileptic at whom everybody laughed, and being able to take case of his little goats, and spreading the good news to mortals: that new times will come, golden times, because in heaven everything is made of gold..., etc.

Although we are looking at two very different illnesses, schizophrenia and epilepsy, both have in common the paranoid syndrome characterized by delusions and hallucinations, in this case with mystical content. It is true that Ricardo Jesus, from his damaged brain, shows himself to us as much simpler and more concrete in the explanation of his contents, but in both – and unlike what happens in mythology – the encounter with God occurs face to face, and in an atmosphere of extreme closeness and familiarity. In the case of Teresa, Jesus appears to her there in the garden or in her own bedroom, and she recognizes him immediately, and she was not subjugated by his light, like Paul of Tars on the way to Damascus. She is even able to describe details of Jesus' face, which appears in front of her, looking at her, unlike Aeneas, Moses or the disciples in Emmaus. But the nearness of the encounter does not stop there: Jesus "shows her all his presence" and, in a certain way merging with her, "he caresses her heart". We psychiatrists know that when a patient suffering from delusions, and especially a schizophrenic one, states something like this, he is not making a metaphor: If he were, we could not speak of delusion. Metaphor is the opposite of delusion. Delusion can be understood precisely as a literal interpretation of the metaphor. Teresa actually experiences the physical sensation that Jesus caresses her heart. And what greater intimacy can exist than the one given with someone who is able to caress our heart? In the case of Ricardo the encounter occurs in heaven itself, where he, magically transformed into Jesus Christ through his name, seats at the right hand of the Father. There he does not experience any worry, any subjugation, or prostration in the presence of the divine. God shows Himself to him in all his glorious splendour, but this does not surprise him, because he himself is a little like god. His resurrection after the series of epileptic seizures allows him an immediate access to the level of the Creator, from which he will come down to bring the good news to men. He is going to enlighten, with that beam radiating from the celestial gold, first his neighbours, in the little village in the mountains where he keeps his herd of goats, and everybody else who is willing to receive it.

In summary, in the realm of madness – and these are not two isolated cases, but rather examples of what is common in such cases – the encounter with God takes place in a way directly opposite to what happens in the myth: whereas in the latter, God manifests himself in his divinity at the moment of the farewell, when leaving ("turning His back") – as Venus shows herself to Aeneas – in madness he appears in a way so direct, immediate and close that the patient is able to reach the total identification with the divine, even wondering if he and God may be the same thing, even though this may cause divinity to lose all its greatness and majesty. "That radiation, that light emanating from the world of the divine, ceases to be perceived in religious delusion", affirms Tellenbach [18], while attempting to describe how these two fundamental roots of human experience, hope and truth, are disfigured and in a certain way destroyed, respectively, in the two main forms of mental illnesses: depression and schizophrenia.

As we appreciate the deformation of the divine that we see occurring on the edge of madness, we understand that in Book Four of The Aeneid the god Mercury, letting Aeneas perplex, can have "...vanished into thin air, far beyond human ken." [1, IV, p.143], but we can also better understand the mysterious words from Yahweh to Moses in Exodus, 33, 20: "...for man cannot see me and live." [2].

## Authors' contributions

This essay is the product of a semester workshop on myth and madness, organized by OD at the Department of Psychiatry of the University of Chile, in which OV was invited to participate, as specialist in ancient philosophy and literature.

OD carried out the analysis of the clinical cases, and the discussion; besides, he provided some of the Bible quotations, the cases of the mystical poets Saint John of the Cross and Saint Theresa of Ávila, and the references to the poetry by Rainer Maria Rilke.

OV provided his deep knowledge on Virgil's Aeneid, Plotinus' philosophy and the Old Testament.

Both authors read and approved the final manuscript.
